# Integrated analysis of competing endogenous RNA networks in peripheral blood mononuclear cells of systemic lupus erythematosus

**DOI:** 10.1186/s12967-021-03033-8

**Published:** 2021-08-21

**Authors:** Wencong Song, Jie Qiu, Lianghong Yin, Xiaoping Hong, Weier Dai, Donge Tang, Dongzhou Liu, Yong Dai

**Affiliations:** 1 Department of Clinical Medical Research Center, Guangdong Provincial Engineering Research Center of Autoimmune Disease Precision Medicine, Shenzhen Engineering Research Center of Autoimmune Disease, The Second Clinical Medical College, Jinan University (Shenzhen Peoples Hospital), Shenzhen, 518020 China; 2 The First Affiliated Hospital, Jinan University, Guangzhou, 510630 China; 3 School of Computer Science and Engineering, Yulin Normal University, Yulin, China; 4 Guangxi Key Laboratory of Metabolic Disease Research, Central Laboratory of Guilin NO. 924 Hospital, Guilin, 541002 China; 5 College of Natural Science, University of Texas at Austin, Austin, TX 78712 USA

**Keywords:** ceRNAs, Integrative analysis, SLE, RNA sequencing

## Abstract

**Background:**

Systemic lupus erythematosus (SLE) is an autoimmune disease with a complicated pathogenesis, and its aetiology has not been clearly unveiled. The lack of effective diagnosis and treatment methods makes it necessary to explore the molecular mechanism of SLE. We aimed to identify some critical signalling pathways and key competing endogenous RNAs (ceRNAs) underlying the molecular mechanism of SLE and to map out the systematic signalling networks by integrating the data on different kinds of RNAs.

**Methods:**

Peripheral blood mononuclear cells (PBMCs) were collected from both SLE patients and healthy subjects, RNA was extracted from the PBMCs, and RNA libraries including ribosomal RNA-depleted strand-specific libraries and small RNA libraries were built for deep RNA sequencing (RNA-seq). RNA-seq yielded differential expression profiles of lncRNAs/circRNAs/miRNAs/mRNAs related to SLE. The DAVID database (v. 6.8) was employed for Gene Ontology (GO) and KEGG pathway analysis. ceRNA networks (circRNA/lncRNA-miRNA-mRNA) were constructed and visualized using Cytoscape software (v. 3.5.0). The TargetScan and miRanda databases were used to predict target relationships in ceRNA networks. qRT-PCR was used to verify our data.

**Results:**

Differential expression of ceRNAs related to SLE was detected in SLE patients’ PBMCs: 644 mRNAs (384 upregulated, 260 downregulated), 326 miRNAs (223 upregulated, 103 downregulated), 221 lncRNAs (79 upregulated, 142 downregulated), and 31 circRNAs (21 upregulated, 10 downregulated). We drew ceRNA signalling networks made up of the differentially expressed mRNAs/miRNAs/lncRNAs/circRNAs mentioned above, and the hub genes included IRF5, IFNAR2, TLR7, IRAK4, STAT1, STAT2, C2, and Tyk2. These hub genes were involved in ceRNA signalling pathways, such as the IL-17 signalling pathway and type I interferon signalling pathway.

**Conclusions:**

We explored the differential expression profiles of various kinds of ceRNAs and integrated signalling networks constructed by ceRNAs. Our findings offer new insights into the pathogenesis of SLE and hint at therapeutic strategies.

**Supplementary Information:**

The online version contains supplementary material available at 10.1186/s12967-021-03033-8.

## Background

Systemic lupus erythematosus (SLE) is a complex systemic autoimmune disease that is characterized by overproduction of autoantibodies against self-antigens, complement consumption, and chronic inflammation [1]. According to statistics updated in September 2016, the prevalence of SLE varies between different areas in the world, and the prevalence could be as high as 241/100,000 people (95% CI 130,352) in specific areas, such as North America [2]. In addition, SLE is a disease with a clear sex tendency that mainly affects women of childbearing age [3].

Multiorgan pathologies caused by immune complex deposition in SLE result in diverse clinical manifestations, such as arthritis and skin disease [4]. Disease heterogeneity and diverse manifestation characteristics make early diagnosis and treatment difficult [5]. The study of aetiology in SLE will improve our understanding of this disease and thus help with the early diagnosis and treatment of the disease.

Multiple factors, including endocrine factors, genetic factors, infections and environmental factors, contribute to the development of SLE, but the exact aetiology of this disease has not yet been clearly unveiled [6, 7].

Many studies have shown that various kinds of RNAs, including coding and noncoding RNAs, contain abnormal expression in SLE, and their distinct expression profiles are correlated with the distinct pathophysiological states in SLE patients. These coding and noncoding RNAs include messenger RNAs (mRNAs), long noncoding RNAs (lncRNAs), microRNAs (miRNAs), and circular RNAs (circRNAs). According to earlier studies, lncRNAs and circRNAs regulate the expression of targeted genes (mRNAs) by competing for the binding of mRNAs to miRNAs; thus, all of these RNAs were defined as competing endogenous RNAs (ceRNAs). Different kinds of ceRNAs can be distinguished by their unique characteristics, such as their length. miRNAs are single-stranded RNAs of 18–22 nucleotides [8] and lncRNAs longer than 200 nucleotides [9], while circRNAs contain closed loops and are much more stable than other kinds of RNAs [10].

### Signalling networks made of ceRNAs

With the development of bioinformatics and omics technologies, an increasing number of ceRNA networks have been identified, which will enrich our understanding of the pathogenesis of SLE. As summarized below, the ceRNA networks include lncRNA–miRNA–mRNA and circRNA–miRNA–mRNA.

For lncRNA–miRNA–mRNA interactions, Ye et al. found a ceRNA network consisting of 155 validated mRNAs, 15 miRNAs and 7 lincRNAs, and the network had a robust or weak “IFN signature”, which was closely related to the pathogenesis of SLE [11].

For circRNA–miRNA–mRNA networks, Tian et al. identified a ceRNA network consisting of mmu_circRNA_34428, miR-338-3p, miR-670-3p, miR-3066-5p, miR-210-5p and their corresponding mRNA targets in an NZB/W F1 lupus nephritis mouse disease model [12]. Zhang et al. identified a ceRNA network made up of hsa_-circ_0012919, miR-125a-3p, CD70 and CD11a in SLE CD4+ T cells [13]. Another study showed that circIBTK in a ceRNA network could inactivate protein kinase B (AKT) by binding to miR-29b in SLE, which impedes the development of SLE [14]. Zhang et al. found that hsa_-circ_0049224 and hsa_-circ_0012919 affect the expression of DMNT1, CD70 and CD11a by sponging miR-125a-3p in SLE CD4+ T cells [13].

Studies also showed that different circRNAs play a different role in specific canonical signalling pathways in SLE. For example, hsa_circ_100226 targeted p65 by regulating miR-138 in the NF-κB inflammation pathway [15], while upregulated circRNA_002453 in the plasma of lupus nephritis (LN) was associated with the severity of renal damage [16].

From this evidence, researchers have recognized some pathways that are closely related to the pathogenesis of SLE, such as JAK-STAT (STAT1, STAT4) [17], oxidation-related superoxide dismutase (SOD), NADPH [18], apoptosis (Fas, Bcl2, TNF and IFN) [19] and complement and coagulation cascades.

ceRNA and ceRNA networks unveiled by former studies help us better understand the molecular pathogenesis of SLE. However, most ceRNA studies in SLE have focussed on a single ceRNA network, such as circRNA/lncRNA–miRNA–mRNAs. Discussion of both circRNA–miRNA–mRNA networks and lncRNA–miRNA–mRNA networks in the same study is rare, not mention to the crosstalk between the two different kinds of ceRNA networks.

To construct ceRNA regulatory networks of SLE, PBMCs were collected from both SLE patients and healthy subjects in this study. RNA was extracted from PBMCs, and then RNA libraries, including ribosomal RNA-depleted strand-specific libraries and small RNA libraries, were built for deep RNA sequencing (RNA-seq). The RNA-seq data between SLE patients and healthy subjects were compared to identify the differential expression profiles of RNAs (lncRNAs/circRNAs/miRNAs/mRNAs), and these RNAs were submitted to integrative analysis. The GO and KEGG pathway databases were utilized for functional and pathway analysis to identify some key RNAs and signalling pathways that play critical roles in the pathogenesis of SLE. Our findings will offer new, deeper insight into the molecular mechanisms of SLE pathogenesis and provide empirical support to therapeutic target investigations for SLE.

## Methods

### Blood samples collection

Blood samples were collected from 40 healthy subjects (H_B) and 40 SLE patients (SL_B) in Shenzhen People’s Hospital (Guangdong, China) from June 2018 to December 2019. All SLE patients and healthy subjects were of Han Chinese ancestry. Inclusion criteria: SLE patients diagnosed with SLE and healthy subjects without SLE or other immune diseases who were not treated with immunosuppressants. The SLE diagnostic criteria in this study were according to the ACR (the 1997 revised criteria of American College Rheumatology) [20]. The SLE classification criteria and the characteristics of all patients are presented in Additional file 7: Table S5. The study was approved by the ethics committee of Shenzhen People's Hospital, and the experiments were carried out under the guidance of the Declaration of Helsinki of 1995. All participants signed informed consent forms.

### PBMCs preparation

Ethylene diamine tetraacetic acid (EDTA)-anticoagulated venous blood samples (5 ml for each donor) were obtained from SLE and healthy patients, and peripheral blood mononuclear cells (PBMCs) were prepared as described in a previous study [10]. Briefly, blood samples were centrifuged at 3200 rpm for 10 min at room temperature. Plasma from blood samples was removed to an RNase-free tube for cryopreservation. PBMCs were obtained from remaining anticoagulated whole blood by using the Ficoll density gradient separation method (Sigma, USA). PBMCs were stored at −80 °C until further treatment.

### Construction and sequencing of small-RNA library

Total RNA was extracted from PBMCs with TRIzol reagent (Invitrogen, CA, USA) or the Total RNA Purification Kit (LC Sciences, Houston, USA). The RNA amount and purity of each sample were quantified using a NanoDrop ND-1000 (NanoDrop, Wilmington, DE, USA). RNA integrity was assessed by a Bioanalyzer 2100 (Agilent, CA, USA) with RIN number > 7.0 (RIN > 8.4 in our experiment, data not shown) and was confirmed by electrophoresis with denaturing agarose gel. TruSeq Small RNA Sample Prep Kits (Illumina, San Diego, USA) were used for small RNA library construction, and then the miRNA library was sequenced by an Illumina HiSeq 2500 with 50-base single-end reads following the vendor's recommended protocol. For more information, please refer to our Additional file 10: Methods-ceRNA analysis protocol).

### Ribosomal RNA-depleted strand-specific library construction and sequencing

After total RNA extraction from PBMCs by TRIzol reagent, construction of two ribosomal RNA-depleted strand-specific libraries was conducted as previously reported [21, 22]. Briefly, the quantity and quality of RNA samples were analysed as described above. Then, RNA was purified by removing ribosomal RNA from total RNA using the Epicentre Ribo-Zero Gold Kit (Illumina, San Diego, USA). After purification, the poly(A)- and poly(A) + RNA fractions were randomly fragmented into small pieces. Finally, the cDNA libraries were constructed by reverse transcription of the sheared products using the mRNA-Seq sample preparation kit (Illumina, San Diego, USA). The average insert size for the final cDNA library was 300 ± 50 bp. Then, cDNA libraries were sequenced on an Illumina NovaSeq™ 6000 (LC-Bio Technology CO., Ltd., Hangzhou, China) with 2 × 150 bp paired-end sequencing (PE150). To find more detailed information on the library construction, please refer to our Additional file 10: Methods-ceRNA analysis protocol).

### Differential expression analysis and identification of RNAs

Different methods were utilized for expression analysis and identification of different kinds of RNAs. For miRNA, an in-house program of ACGT101-miR (LC Sciences, Houston, TX, US) or Cutadapt [23] was used to remove sequencing adaptors, low-quality bases and undetermined bases from the raw sequencing datasets. For other kinds of RNAs (mRNAs, circRNAs, lncRNAs), fastp was utilized (Additional file 10: Methods-ceRNA analysis protocol).

Then, the quality of expressed sequence tags was estimated using FastQC, and the valid reads were mapped to the human reference genome (GRCh38) by Bowtie 2 [24] and TopHat2 [25]. The differential expression levels of mRNAs/miRNAs/lncRNAs were calculated with StringTie [26] and Fisher’s exact test (F-test). circRNA expression levels from different samples or groups were calculated by SRPBM. Expression data with |log2 (fold change) |> 1 and p-value < 0.05 were defined as statistically significant. The value setting of |log2 (fold change) |> 1 for the threshold of fold change for RNAs can generate enough RNA candidates. Detailed information is provided in the Additional file 10: Methods-ceRNA analysis protocol.

### Functional enrichment analysis

By volcano plot filtering and GO (http://www.geneontology.org) and KEGG analyses (http://www.genome.jp/kegg), the biological functions and pathways of the candidate target genes involved in ceRNA networks (lncRNAs/circRNAs-miRNAs-mRNAs) were predicted. Then, the ceRNA network-related target genes were put into the Database for Annotation, Visualization and Integrated Discovery (DAVID, v. 6.8) for GO and KEGG pathway analysis. Each GO and KEGG pathway term with a p value < 0.05 and |log2(fold change)|> 1 was defined as significant.

### ceRNA network construction

TargetScan (v. 5.0) and miRanda (v. 3.3a) [27] were used to predict the potential targeting relationship of miRNA-mRNA (3′UTR) pairs and miRNA-lncRNA/circRNA pairs. UTR sequence data were acquired from Ensembl (http://ensemblgenomes.org). The regulatory networks of interest in our study included lncRNA-miRNA, miRNA-mRNA, circRNA-miRNA, lncRNA-miRNA-mRNA and circRNA-miRNA-mRNA in SLE PBMCs. We set the context score cut-off as ≥ 50 in TargetScan and max energy to < −10 in miRanda, and a p value < 0.05 indicated significant binding possibilities between ceRNAs. Then, the overlapping predictions between the two programs were considered effective target pairs. All the ceRNA networks were drawn by Cytoscape (v. 3.5.0) [28].

### qRT-PCR analysis

Quantitative real-time polymerase chain reaction (qRT-PCR) was used to verify the identified sequencing data of circRNAs/lncRNAs/mRNAs/miRNAs from PBMCs of both H_B and SL_B.

To obtain miRNAs, total RNA was extracted from the PBMCs described above and subjected to first-strand cDNA synthesis using the TRUEscript 1st Strand cDNA SYNTHESIS Kit (Aidlab, Beijing, China). The specific primers used in the qRT-PCR are listed in Additional file 8: Table S6. qRT-PCR was performed using a SYBR PrimeScript miRNA RT-PCR Kit (TianGen Biotech, Beijing, China). Each qRT-PCR mix was referenced from a previous study, with minor modifications: 5 μl 2 × SYBR® Green Supermix, 0.5 μl specific forward primer, 0.5 μl specific reverse primer, 1 μl cDNA template and 3 μl ddH2O. The qRT-PCR programme was as follows: 95 °C for 3 min followed by 39 cycles of 95 °C for 10 s and 60 °C for 30 s, with a melt curve analysis. The experiment was performed in a real-time PCR system (analytikjena-qTOWER2.2, Germany). U6 was used as an internal control gene for miRNAs. Three independent biological replicates were run for each gene. The qRT-PCR data were analysed by the comparative 2-ΔΔCt method [29], and the average of three independent experiments for each gene was calculated as the relative expression level by GraphPad Prism (version 8.0.1).

To obtain lncRNAs and mRNAs, we used a PrimeScript™ RT Reagent Kit (Perfect Real Time, Takara, Japan) to prepare cDNA. The qRT-PCR was conducted as described above. GAPDH expression was used as the endogenous control for normalization of sample data.

To obtain circRNA [10], extracted total RNA was reverse-transcribed to cDNA with random primers using a RevertAid™ First-Strand cDNA Synthesis Kit (Fermentas, Waltham, MA). Real-time quantitative PCR was performed as described above. Divergent primers were designed for circRNAs (Additional file 8: Table S6). We used GAPDH as an internal control and calculated the relative gene expression with the 2-ΔΔCt comparative threshold cycle method.

## Results

### The expression profile of mRNAs/miRNAs/lncRNAs/circRNAs in SLE

**Fig. 1:**
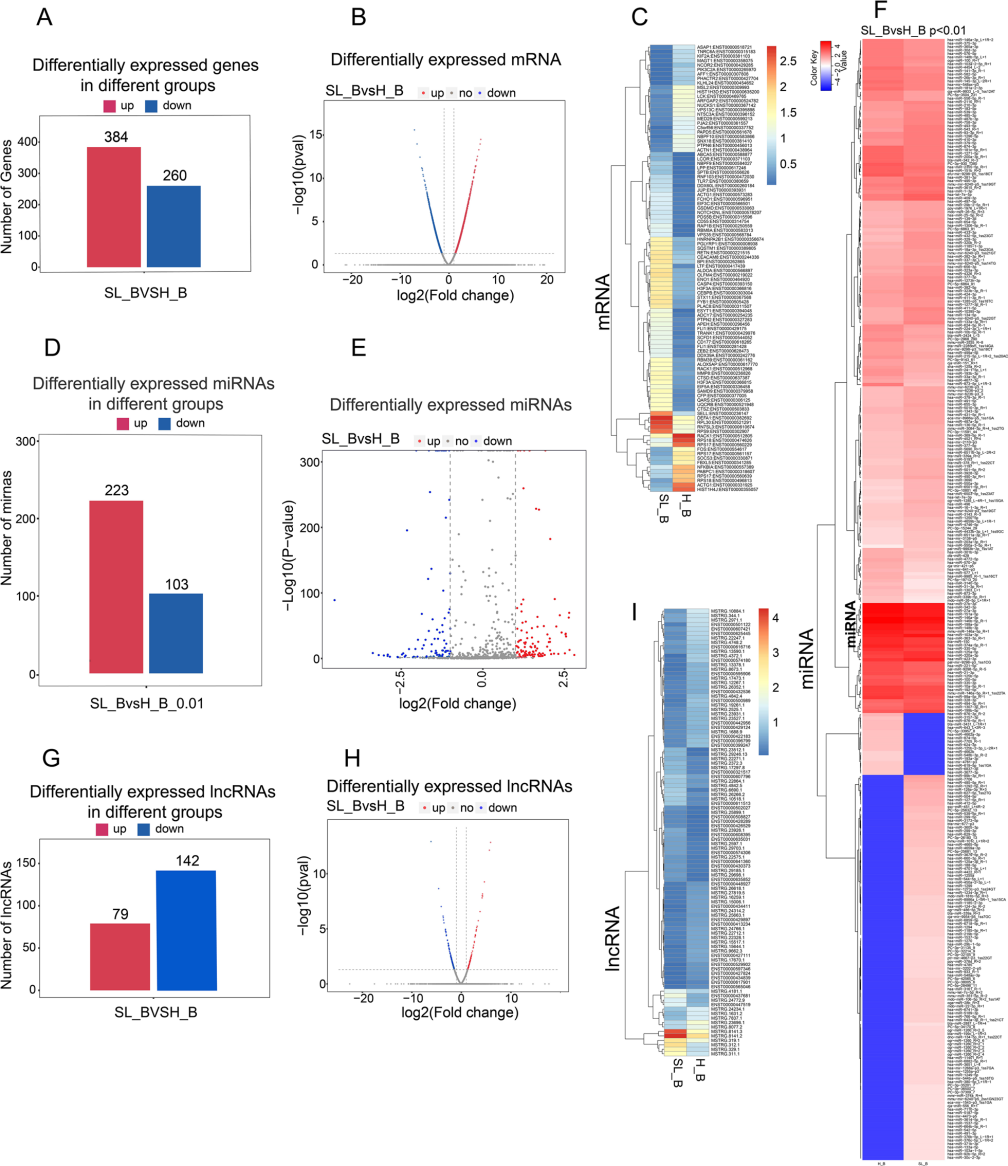
The expression profile of mRNA/miRNA/lncRNA in PBMCs. **A**–**C** Differentially expressed genes (mRNAs) in SLE patients. The number of genes with distinct expression in SLE patients is summarized and presented in bar charts (**A**) and volcano plots (**B**). The horizontal axis presents the log2(fold change), which shows the fold change of each differentially expressed gene in SLE patients vs. healthy controls. The vertical axis displays the significance level of differential gene expression calculated with the −log10(p-value). The blue dots represent significantly upregulated genes, while red dots characterize the considerably downregulated genes. The grey dots denote that the expression level of genes was not striking. Data are shown if they had (|log2 fold change|≤ 2 and P-value > 0.05)). (C) Heatmap of differentially expressed genes between the healthy control (H_B) and SLE patient venous blood (SL_B) in detail. Differential expression was defined as |log2 fold change|≥ 2 and P-value < 0.05. **D**–**F** Differential expression profile of miRNAs between H_B and SL_B. **D** Bar chart showing the differential expression profile of miRNAs in H_B and SL_B under the filter threshold (p < 0.01). **E** The differential expression profile of miRNAs between SL_B group and H_B is presented in a volcano plot (p < 0.01). **F** Heatmap presenting the differentially expressed miRNAs between H_B and SL_B in detail with filter threshold p < 0.01. **G**–**I** Differential expression profile of lncRNAs between groups of H_B and SL_B. Differential expression profiles of lncRNAs are shown in (**H**) volcano plots and bar chart (**G**). The details of the differentially expressed lncRNAs between H_B and SL_B are presented in the heatmap (**I**)

To explore the potential functions of mRNAs/miRNAs/lncRNAs/circRNAs in SLE, we analysed the expression profiles of these RNAs. From the transcriptional data, 644 transcripts were differentially expressed; 384 genes were upregulated, while 260 were downregulated (Fig. 1A, Additional file 3: Table S1). Further analysis showed that among all the differentially expressed transcripts, 99 were significant (60 genes upregulated, 39 downregulated) (Fig. 1B). The details of the differential expression of mRNAs are shown in a heatmap (Fig. 1C).

For miRNAs, the data are presented in a bar graph that summarizes the data for the differential expression profile of miRNAs in SL_B and H_B under the filter threshold (p < 0.01) (Fig. 1D, Additional file 4: Table S2). Detailed miRNA information is presented in the heatmap (Fig. 1F). When compared to H_B, SL_B contained 223 upregulated miRNAs and 103 downregulated miRNAs (p < 0.01). These data are also presented in volcano plots (Fig. 1E).

For lncRNAs, 221 differentially expressed lncRNAs were identified in our study. Among the differentially expressed lncRNAs, 79 lncRNAs were upregulated, 142 downregulated (Fig. 1G, Additional file 5: Table S3). The results are shown in volcano plots (Fig. 1H), and some top differentially expressed lncRNAs are shown in the heatmap (Fig. 1I).

**Fig. 2:**
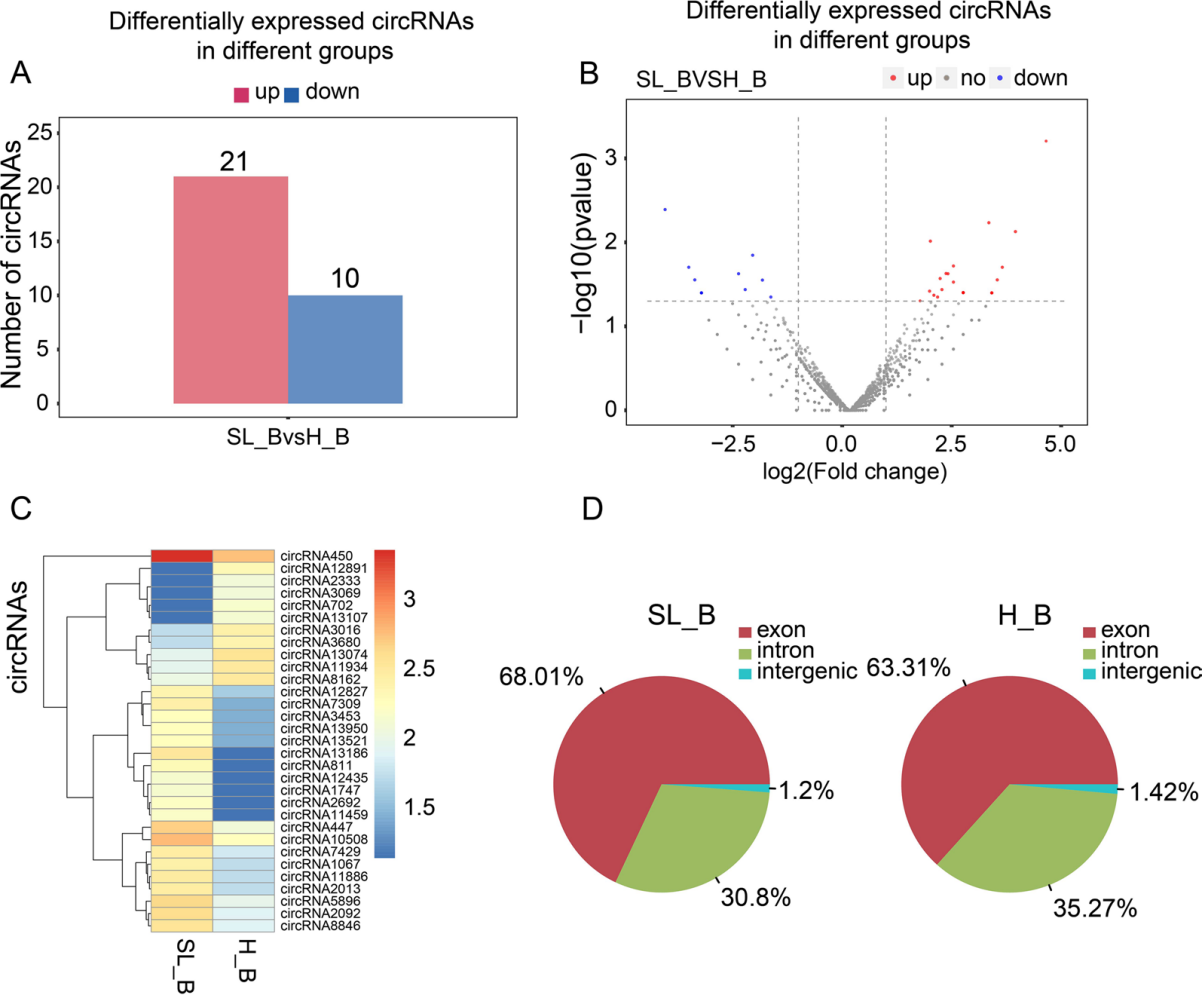
The differential expression of circRNA in PBMCs between H_B and SL_B. The differential expression of circRNA between H_B and SL_B is shown in (**A**) a bar chart, (**B**) volcano plots and (**C**) a heatmap. The source of circRNAs from the genome is presented in the pie chart (**D**)

circRNA differential expression between H_B and SL_B is shown in the volcano plots, bar chart and supplemental file (Fig. 2A–B, Additional file 6: Table S4), and some of the significant ones are presented in the heatmap (Fig. 2C). According to the data, there were 21 upregulated circRNAs and 10 downregulated circRNAs in SL_B. Compared to the H_B, there were more circRNAs in SL_B generated from exons of the genome (Fig. 2D). In contrast, more circRNAs in H_B (35.27%) than in SL_B (30.8%) came from introns.

### GO enrichment and KEGG pathway enrichment analysis for the targeted genes in the ceRNA network

**Fig. 3:**
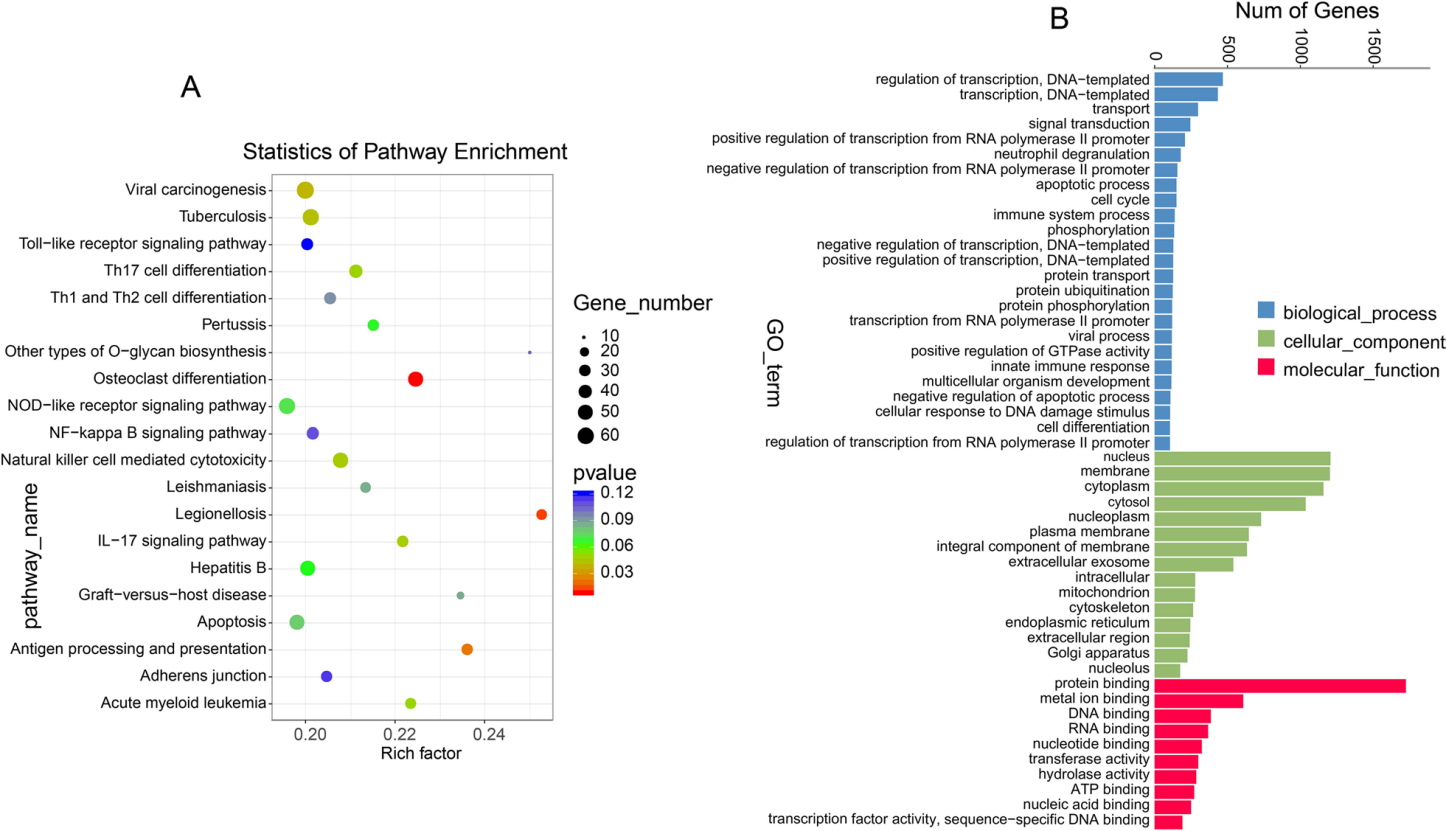
GO enrichment and KEGG pathway enrichment analysis for the targeted genes in the ceRNA network. **A** KEGG enrichment analysis for the differentially expressed targeted genes in the ceRNA network. The horizontal axis indicates the enrichment factor, which represents the enrichment level of differentially expressed genes. The vertical axis displays the regulatory pathways involved in SLE pathogenesis. **B** GO analysis of the differentially expressed targeted genes in the ceRNA network. The horizontal axis indicates the names of hierarchical clusters in cellular component (CC), biological process (BP) and molecular function (MF). The vertical axis displays the number of targeted genes in a particular hierarchical cluster. The GO terms were sorted by the thresholds of mRNA fold change and enrichment level (|log2 fold change |≥ 2 and P-value < 0.05)

Gene Ontology (GO) was conducted for hub gene functional analysis (Fig. 3B). Hierarchical clustering of hub genes according to biological process (BP), molecular function (MF) and cellular component (CC) categories of the GO nomenclature, with screening criteria of p value < 0.05 and |log2 (fold changes) |≥ 1, was conducted. Twenty-five BP, 10 MF and 15 CC GO terms were found. BP terms included apoptotic process, immune system process, negative regulation of apoptotic process, cellular response to DNA damage stimulus, cell differentiation and others. CC included mitochondrion and other GO terms. MF contained ATP binding.

To reveal pathways involving the differentially expressed genes targeted by miRNAs in SLE, KEGG pathway analysis was performed (Fig. 3A). KEGG pathway analysis showed that some pathways participated in the development of SLE, such as cell apoptosis, inflammation pathways (Toll-like receptor signalling pathway, NF-kappa B signalling pathway and NOD-like receptor signalling pathway), and Th17 cell differentiation. Our results also indicated that the genes targeted by miRNAs function in SLE partially through apoptosis-, cell differentiation- and inflammation-related pathways.

### Regulation networks of lncRNA-miRNA, circRNA-miRNA and miRNA-mRNA

**Fig. 4:**
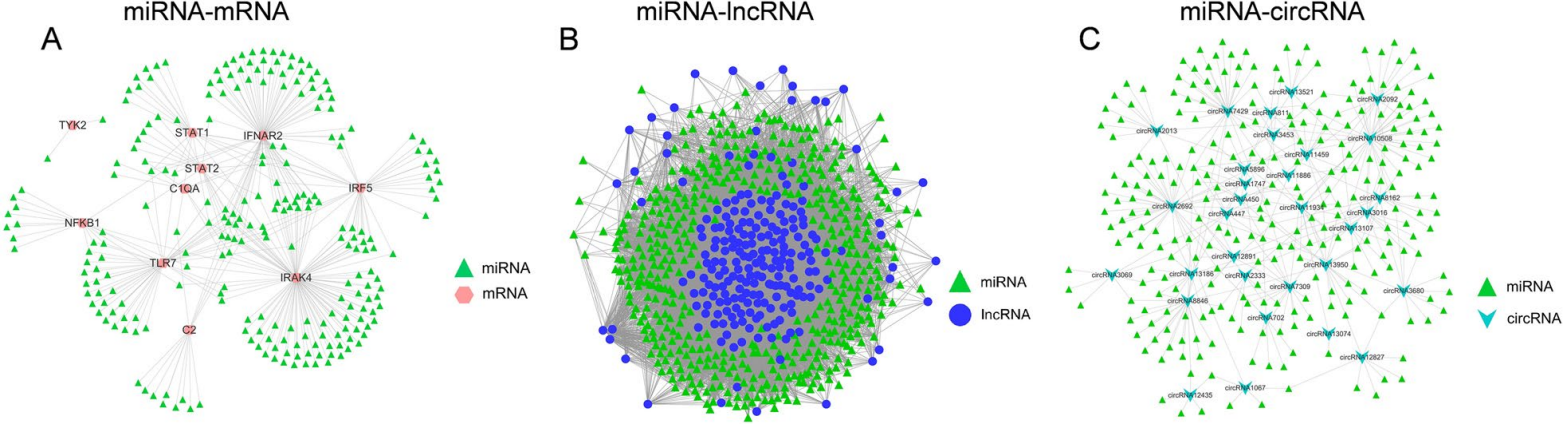
The ceRNA networks consist of two types of RNAs. **A** miRNA-mRNA networks, **B** miRNA-lncRNA networks, **C** miRNA-circRNA networks

When we examined the details of the ceRNA network, we found that the lncRNA/circRNA/miRNA/mRNA network could be divided into three subsets of interaction networks. The subsets of the network were distinguished by the correlation between two different types of RNAs, such as miRNA-mRNA, miRNA-lncRNA and miRNA-circRNA networks (Fig. 4A–C).

### Regulatory network for lncRNAs/miRNAs/mRNAs and circRNAs/miRNAs/mRNAs

**Fig. 5:**
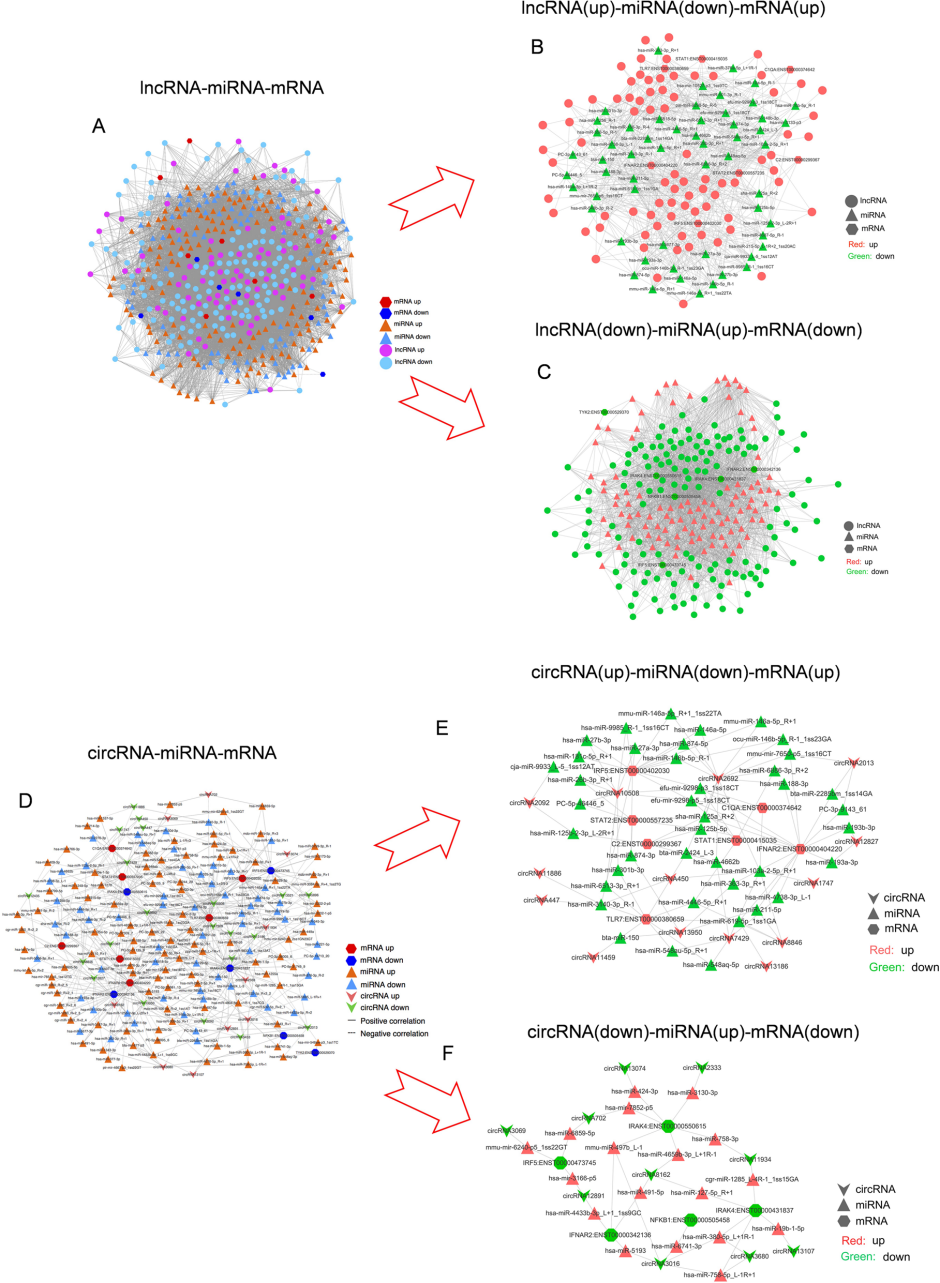
Regulatory network of lncRNAs-miRNAs-mRNAs and circRNAs-miRNAs-mRNAs. **A** Summarization of the lncRNA-miRNA-mRNA signalling network. **B** lncRNA(up)-miRNA(down)-mRNA(up) signalling network. **C** lncRNA(down)-miRNA(up)-mRNA(down) signalling network. **D** Summarization of the circRNA-miRNA-mRNA signalling network. **E** circRNA(up)-miRNA(down)-mRNA(up) regulatory network. **F** circRNA(down)-miRNA(up)-mRNA(down) regulatory network

The interaction network summarized for lncRNA-miRNA-mRNA (Fig. 5A) showed that 6 of 13 of the top differentially expressed mRNAs were downregulated, and the 6 downregulated mRNAs correlated with 105 upregulated miRNAs and 141 downregulated lncRNAs in this regulated network (Fig. 5B).

At the same time, 7 mRNAs were upregulated, and these 7 upregulated mRNAs correlated with 57 downregulated miRNAs and 79 upregulated lncRNAs in this regulatory network (Fig. 5C).

The interaction network summarized for ceRNAs (circRNA-miRNA-mRNA) (Fig. 5D) contained 5 of the top differentially expressed and downregulated mRNAs, 18 upregulated miRNAs and 10 downregulated circRNAs in this regulated network (Fig. 5E).

At the same time, 7 mRNAs were upregulated, which correlated with 40 downregulated miRNAs and 14 upregulated circRNAs in this regulatory network (Fig. 5F).

### ceRNA networks analysis by integrative method

**Fig. 6:**
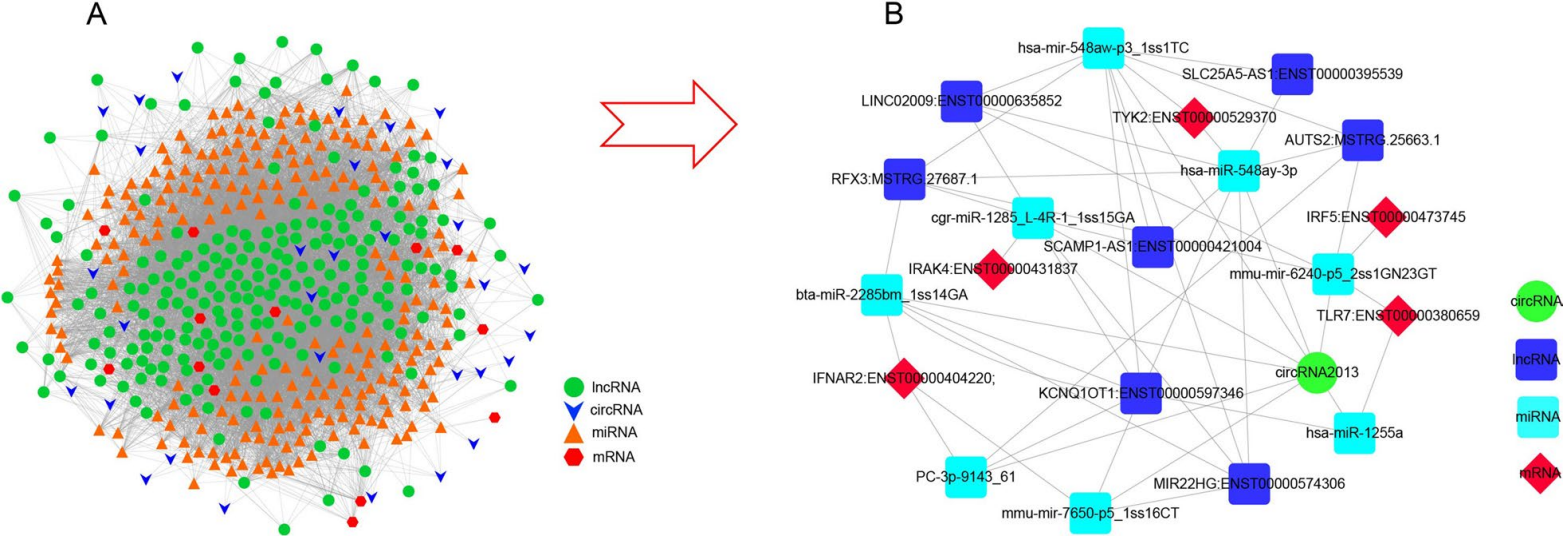
ceRNA signalling networks of lncRNAs/circRNAs-miRNAs-mRNAs. **A** Summarization of the interaction ceRNA network for lncRNAs/circRNAs-miRNAs-mRNAs. **B** An example of the integration of ceRNA signalling networks

The interaction network was summarized for ceRNAs (lncRNA/circRNA-miRNA-mRNA) (Fig. 6A–B). In the ceRNA network, there were 13 top differentially expressed mRNAs (DE-mRNAs), 31 differentially expressed circRNAs, 240 differentially expressed miRNAs and 221 differentially expressed lincRNAs.

A specific regulatory network including four types of ceRNAs (lncRNA/circRNA/miRNA/mRNA) was identified in our study. This regulatory network was made up of 1 circRNA, 5 mRNAs, 7 lncRNAs, and 8 miRNAs. Several hub genes identified in this ceRNA network have been reported previously, such as Tyk2 [30], TLR7 [31], and IRF5 [32].

### Data validation by RT-PCR

The validation of RNA sequencing data was conducted by RT-PCR. A panel of 4 genes (mRNAs), 2 circRNAs, 2 lncRNAs and 2 miRNAs were selected from among the most differentially expressed genes. Our technical validation data (Additional file 1: Figure S2) showed that circRNA2013 was downregulated in SLE patient PBMCs, while in our RNA sequencing data, it was upregulated 5.35-fold (FC), which was contrary to our validated RT-PCR data. The expression level of circRNA3016 was lower in SLE patients than in control subjects in both sequencing and RT-PCR data; nevertheless, the data were significant only in the sequencing data and not in RT-PCR validation result. lncRNA-LIN02009 showed significantly higher levels in the sequencing data from SLE patients’ PBMCs, which was consistent with our RT-PCR data. Another lncRNA, SLC25A5-AS1, shared a similar expression trend in both sequencing and RT-PCR data: lower expression in SLE patient PBMCs than in controls, but the difference in the RT-PCR data was not significant. For microRNA RNA sequencing data validation, both sha-miR-125a_R + 2 and pc-3p-9143_61 were downregulated in SLE patients, but neither one was significant by RT-PCR. Among messenger RNAs, the C1QA gene was upregulated in PBMCs of SLE patients, while TYK2 was downregulated in both their RT-PCR and sequencing data. The expression level of STAT2 (ENST00000557235) presented a similar trend between the RT-PCR and sequencing data, while the expression of C2 (ENST00000299367) in our RT-PCR results was opposite to the sequencing result. In the sequencing data, C2 was 4.99-fold higher in SLE subjects than in healthy controls, while it presented a downregulated trend in our RT-PCR validation data.

## Discussion

SLE is an autoimmune disease with complicated pathogenesis and aetiology. The existing treatment methods, including immunosuppressive agents and new therapeutic interventions, have increased the survival rate of SLE patients. Nevertheless, both the main molecular mechanisms of immune dysfunction and the key driver of pathogenesis in SLE remain unclear. Therefore, there are still some challenges in both the early diagnosis and individualized treatment for this disease [33].

Abnormal epigenetic regulation has been reported previously and potentially plays an important role in the pathogenesis of SLE, and ceRNAs are important forms of epigenetic modifications [34]. In our study, we used RNA-seq to detect differential expression levels of lncRNAs, circRNAs, miRNAs and mRNAs in both SLE patients and healthy individuals. We discovered some hub genes and disease-related signalling pathways, such as IRF5, IFNAR2, TLR7, IRAK4, STAT1, STAT2, C2, and Tyk2 and their pathways, and signalling networks, such as circRNA2692-miRNA125b-stat2, circRNA2692-miRNA125b-C2, circRNA2013-PC-3P-9143_61-IFNAR2, and circRNA2013-hsa-mir-6741-nfkb1.

By KEGG and GO enrichment analysis and integrated methods, some regulatory pathways containing hub genes and related ceRNA signalling networks are presented in Table 1.

**Table 1:** Summary of KEGG and GO analyses of ceRNAs

Investigation items list	Name of KEGG pathways and GO terms
GO terms identified by previous studies	Type I interferon signaling pathway, Regulation of lymphocyte activation, Cytokine-mediated signaling pathway, B cell activation, Response to oxidative stress, T cell receptor signaling pathway
Partial GO terms identified by our current study	Regulation of transcription, DNA-Templated, Transcription, DNA-Templated, Transport, Signal transduction, Apoptotic process, Cell cycle, Immune system process, Phosphorylation
Pathways identified by previous studies	Jak-STAT signaling pathway, Complement and Coagulation Cascades, Apoptosis, NF-kappa B signaling pathway TNF signaling pathway, T cell receptor signaling pathway Systemic lupus erythematosus, Toll-like receptor signaling pathway, IL-17 signaling pathway
Partial pathways identified by our current study	Toll-like receptor signaling pathway, NOD−like receptor signaling pathway, NF−kappa B signaling pathway, IL−17 signaling pathway, Apoptosis, Acute myeloid leukemia
Intersection of GO terms and KEGG pathways from previous studies and this study	Type I interferon signaling pathway, Jak-STAT signaling pathway, PI3K-Akt signaling pathway, NF-kappa B signaling pathway TNF signaling pathway, T cell receptor signaling pathway, Systemic lupus erythematosus, Toll-like receptor signaling pathway, Apoptosis
Intersection of GO terms and KEGG pathways from previous studies and the top 30 of this study	Jak-STAT signaling pathway, Apoptosis, NF-kappa B signaling pathway, TNF signaling pathway, Toll-like receptor signaling pathway, IL-17 signaling pathway
Intersection of significant GO terms and KEGG pathways from previous studies and this study	IL-17 signaling pathway, Type I interferon signaling pathway

### ceRNAs identified in previous studies

lncRNAs such as linc0949 and linc0597 [35], GAS5, lnc7074, lnc0640, lnc5150, NEAT-1, MALAT1 and lnc-DC [36–38] have a potential role in the pathogenesis of SLE. Among them, GAS5, NEAT-1, and MALAT-1 were verified by our study (Additional file 5: Table S3).

Some circRNAs identified in previous studies have potential functions in the pathogenesis of SLE, such as hsa_circ_0045272 [39], hsa_circ_0049224, hsa_circ_0049220 [40], hsa_circRNA_001308, and hsa_circRNA_407176 [10]. In our study, we also unveiled several new circRNAs in SLE, such as circRNA 2092, circRNA 2013, and circRNA 3016 (Additional file 6: Table S4). Some new identical circRNAs in this study have not been reported in SLE studies, but they were demonstrated to function in other biological processes. For example, circRNA 2092 plays a significant role in the development of secondary hair follicles [41].

Some miRNAs related to SLE were also recognized in both previous studies and our current study (Additional file 4: Table S2): miR-126, miR-21, miR-146a, miR-451a, miR-148a, miR-873, miR-1246, miR-25, miR-302, miR-93, miR-9b, miR-22, miR-151a-3p and miR-766-3p [42].

mRNAs acknowledged by previous reports to be associated with the pathogenesis of SLE include stat4, Itgax, Lyn, Tnfaip3 Cdkn1b, Intergenic, Ptpn22, Trex1, Hla-a, Ifi44, Ifi44l, Ifit1, Ly6e and Irf7 [43]. Interestingly, these genes were well represented in the current study (Additional file 3: Table S1).

### The pathways involved in SLE

**Fig. 7:**
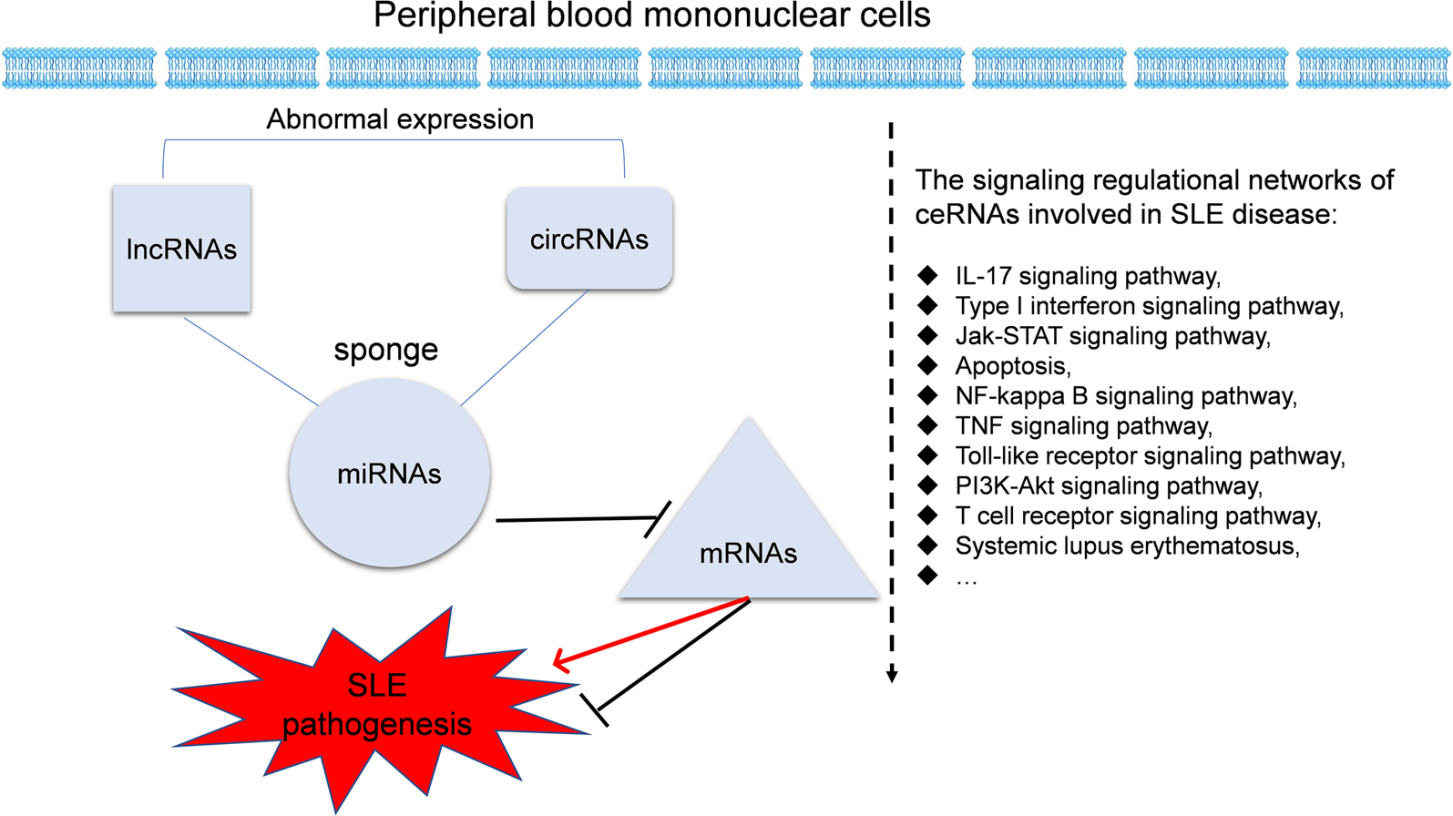
The whole picture of ceRNA regulatory signalling networks in SLE. The ceRNA regulatory networks in SLE were summarized by integrative analysis

By using the KEGG and GO databases, 8562 KEGG pathways and GO terms were discovered in this study. We also summarize 16 frequently mentioned KEGG pathways and GO terms from previous studies (Fig. 7, Table 2). Some of the KEGG pathways and GO terms identified in both previous studies and our current study are shown in Table 1. At the same time, common elements of GO terms and KEGG pathways between the earlier studies and this study are also summarized (Table 1, Additional file 1: Figure S1).

**Table 2:** Frequently mentioned KEGG pathways and GO terms from previous studies

Investigation items list	Name of KEGG pathways and GO terms
GO terms identified by previous studies	Type I interferon signaling pathway Regulation of lymphocyte activation B cell activation Macrophage activation Positive (Negative) regulation of cytotoxic T cell differentiation, IL2 transcription
KEGG Pathways identified by previous studies	Jak-STAT signaling pathway Complement and Coagulation Cascades Apoptosis NF-kappa B signaling pathway PI3K-Akt signaling pathway TNF signaling pathway T cell receptor signaling pathway Systemic lupus erythematosus Toll-like receptor signaling pathway IL-17 signaling pathway

As discussed by previous studies, there are many pathways that play critical roles in the pathogenesis of SLE, such as IL-17 signalling [44], the Jak-STAT pathway [44], type I interferon signalling [27], apoptosis [45], the NF-kappa B pathway [46], the TNF signalling pathway [47], and the Toll-like receptor signalling pathway [48].

The KEGG pathways identified by our current study included cell apoptosis, inflammation pathways (Toll-like receptor signalling pathway, NF-kappa B signalling pathway and NOD-like receptor signalling pathway), Th17 cell differentiation, apoptosis, Th1 and Th2 cell differentiation, TNF signalling pathway, Jak-STAT signalling pathway, natural killer cell mediated cytotoxicity, antigen processing and presentation, T cell receptor signalling pathway, B cell receptor signalling pathway, etc.

Some SLE-related GO terms identified by here include apoptotic process, immune system process, negative regulation of apoptotic process, DNA damage stimulus, cell differentiation, innate immune response, T cell homeostasis, regulation of B cell differentiation, etc.

### Summary of overlapping pathways between earlier studies and ours

ceRNA networks modify or control the expression of key genes in many diseases. For instance, ceRNA network analysis has been mostly used in cancer research [49]. With ceRNA studies extending to autoimmune diseases, many important molecules and key signalling networks have been unveiled, such as the Jak-STAT signalling pathway [17], complement and coagulation cascades [50], and apoptosis[45]. The pathways above are summarized in Additional file 9: Table S7.

Several significant ceRNA-related pathways were unveiled by our current study, such as osteoclast differentiation, legionellosis, antigen processing and presentation, natural killer cell-mediated cytotoxicity, the IL-17 signalling pathway, Th17 cell differentiation, and acute myeloid leukaemia.

Some common pathways identified by our study and earlier ones were identified. For example, the IL-17 signalling pathway [44] and type I interferon signalling pathway [51] were identified in both previous reports and our study, which strongly suggests that these two pathways might play key roles in the pathogenesis of SLE. On the other hand, some pathways recognized by our study have not been well studied before, and these new pathways might provide new insight for aetiology studies of SLE. The ceRNAs identified by our study also add candidates for potential diagnostic biomarker screening.

As more research has been conducted on ceRNAs, a number of genes (molecules) and regulatory pathways that potentially play important roles in the development of SLE have been found. Earlier studies in ceRNA networks were mostly focussed on single regulatory pathways, such as circRNA–miRNA–mRNAs or lncRNA–miRNA–mRNAs. There were no reports discussing the possible relationship between circRNA–miRNA–mRNA sets and lncRNA–miRNA–mRNA sets. Our study conducted an integrated analysis of the ceRNA network for lncRNAs/circRNAs–miRNAs–mRNAs, and some important ceRNA networks were identified. For example, miRNAs (PC-3p-9143_61, hsa-miR-548ay-3p,) lncRNAs (LINC02009) and circRNA 2013 were found in the same regulatory network and correlated with the hub gene TYK2.

Therefore, our ceRNA network study of PBMCs from SLE patients recognized some new ceRNAs and signalling pathways, which will promote the identification of potential diagnostic biomarkers and ceRNA-related regulatory pathways in SLE. Our findings will help us better understand the pathogenesis of SLE, which will aid in research on therapeutic targets.

## Limitations of this article

First, methodological flaws: the sample size of the SLE subjects and healthy controls was relatively small. Larger samples are needed to improve the statistical validity of future studies. Another potential shortcoming is the lack of detailed patient selection criteria and descriptions of their clinical characteristics. SLE is a complex autoimmune disease with a highly varied clinical presentation, and the pathogenic mechanisms involved in SLE are varied. Therefore, more precise criteria, such as activity markers (C3, C4, dsDNA), should be adapted in future studies rather than merely pooling all the patients together for analysis. Finally, the validation methods should be expanded. The hub ceRNAs (genes) were identified by RNA-seq and verified by RT-PCR. Various kinds of biotechnologies for testing PBMCs are needed for further verification of biomarkers, such as ELISA and western blotting.

## Conclusions

In conclusion, this integrated analysis of ceRNA networks identified several hub ceRNAs (genes) and signalling pathways associated with the pathogenesis of SLE. By combining some important signalling pathways and genes identified by previous studies and our study, we have identified some hub genes, KEGG pathways and GO terms that may play critical roles in SLE development. The hub genes and ceRNAs recognized in our study included mRNAs (IRF5, IFNAR2, TLR7, STAT1, C2, Tyk2) and some circRNAs, miRNAs and lncRNAs. The pathways identified here included the Jak-STAT signalling pathway, apoptosis, NF-kappa B signalling pathway, TNF signalling pathway, Toll-like receptor signalling pathway, IL-17 signalling pathway, and type I interferon signalling pathway. Notably, the IL-17 signalling pathway and type I interferon signalling pathway were two of the most significant pathways shown in our study (Fig. 7).

## Supplementary Information

**Additional file 1: Figure S1.** Overlapping common signalling pathways are shown in a Venn diagram. Overlapping of KEGG pathways and GO terms was analysed by comparing both the KEGG and GO terms from previous studies, our current study and some of the significant terms in our current study.

**Additional file 2: Figure S2.** Validation of RNA sequencing data by RT-PCR. Four genes, 2 circRNAs, 2 lncRNAs and 2 miRNAs were analysed by qRT-PCR, and their relative expression levels were normalized to the housekeeping gene GAPDH or U6. Data are represented as mean ± SEM. *p < 0.05 and **p < 0 01 indicate that the differential expression of genes was significant, while ns indicates no significance (p ≥ 0.05).

**Additional file 3.** SL_B vs H_B_transcripts-mRNAs

**Additional file 4.** SL_B vs H_B differential expression of miRNAs

**Additional file 5.** SL_B vs H_B_lncRNA_differential_expression

**Additional file 6.** SL_B vs H_B_circRNA_differential_expression

**Additional file 7: Table S5.** Clinical data of participants.

**Additional file 8: Table S6.** The primers using for PCR amplification.

**Additional file 9.** Common elements of GO terms and KEGG pathways

**Additional file 10.** Protocols for RNA extraction, library construction, sequencing and Bioinformatics analysis

## Data Availability

The raw data for small RNA sequencing and ribosomal RNA-depleted sequencing in our study are available from [Gene Expression Omnibus: GSE146410], but restrictions apply to the availability of these data, which were used under license for the current study, and so are not publicly available.

## Abbreviations

SLE: Systemic Lupus Erythematous
PBMCs: Peripheral blood mononuclear cells
IFN: Interferon
SOD: Superoxide dismutase
lncRNA: Long non-coding RNAs
mRNA: Messenger RNA
circRNAs: Circular RNAs
miRNAs: MicroRNAs
qRT-PCR: Quantitative real-time polymerase chain reaction
BP: Biological processes
MF: Molecular functions (MF)
CC: Cellular components
GO: Gene ontology
KEGG: Kyoto encyclopaedia of genes and genomes
RNA-seq: RNA-sequencing
DAVID: Database for Annotation, Visualization, and Integrated Discovery
AKT: Activate protein kinase B
DMNT1: DNA methyltransferase 1
NADPH: Nicotinamide adenine dinucleotide phosphate
SLEDAI: Systemic Lupus Erythematosus Disease Activity Index
ESR: Erythrocyte sedimentation rate
EDTA: Ethylene Diamine Tetraacetic Acid
