# Supplementary figures and images for: Integrated analysis of competing endogenous RNA networks in peripheral blood mononuclear cells of systemic lupus erythematosus
Source: J Transl Med. 2021 Aug 21;19:362. doi: 10.1186/s12967-021-03033-8 (PMC8380341; doi:10.1186/s12967-021-03033-8)

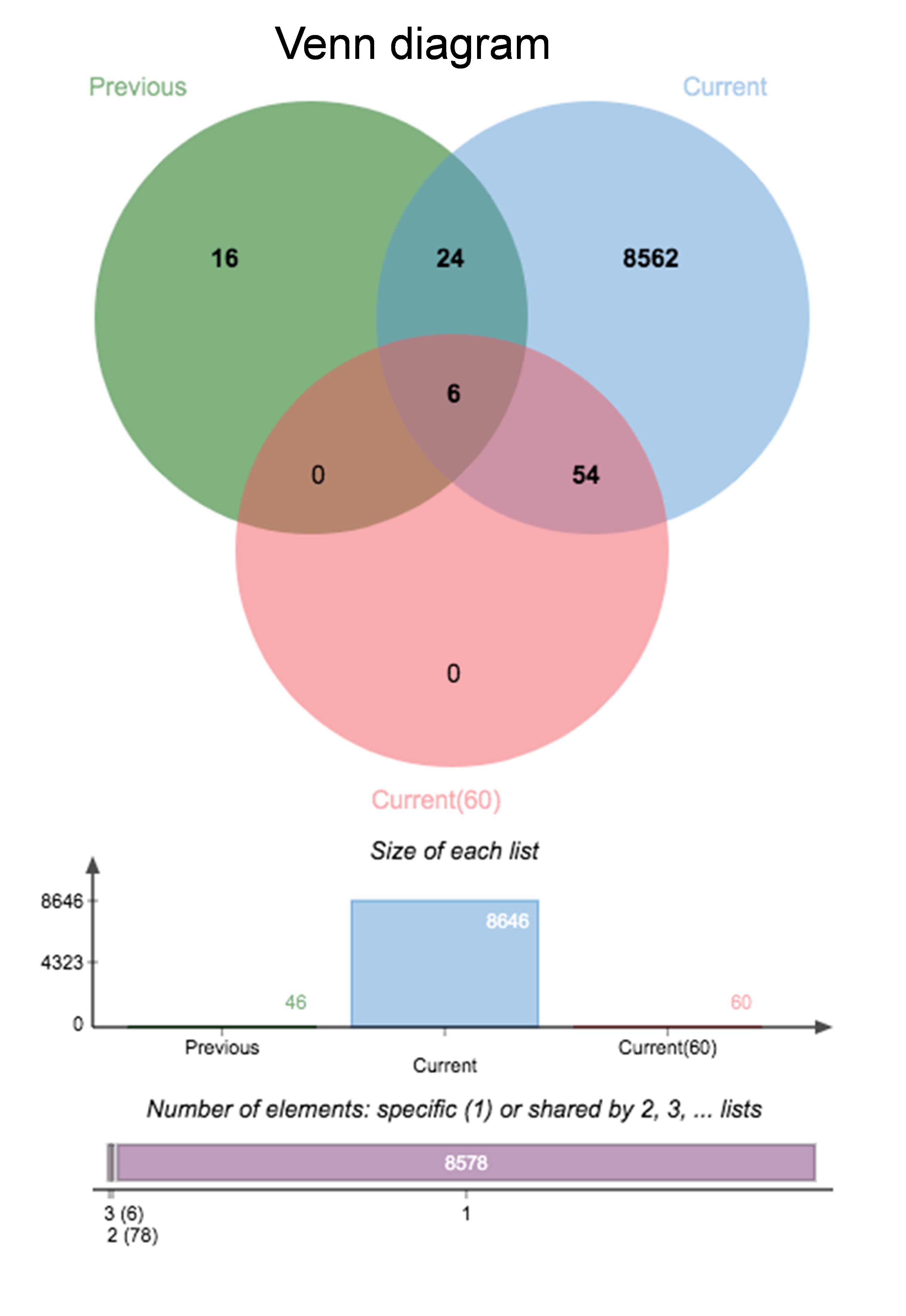

Supplement: Supplementary file 1 — Additional file 1: Figure S1. Overlapping common signalling pathways are shown in a Venn diagram. Overlapping of KEGG pathways and GO terms was analysed by comparing both the KEGG and GO terms from previous studies, our current study and some of the significant terms in our current study. [file 12967_2021_3033_MOESM1_ESM.jpg]

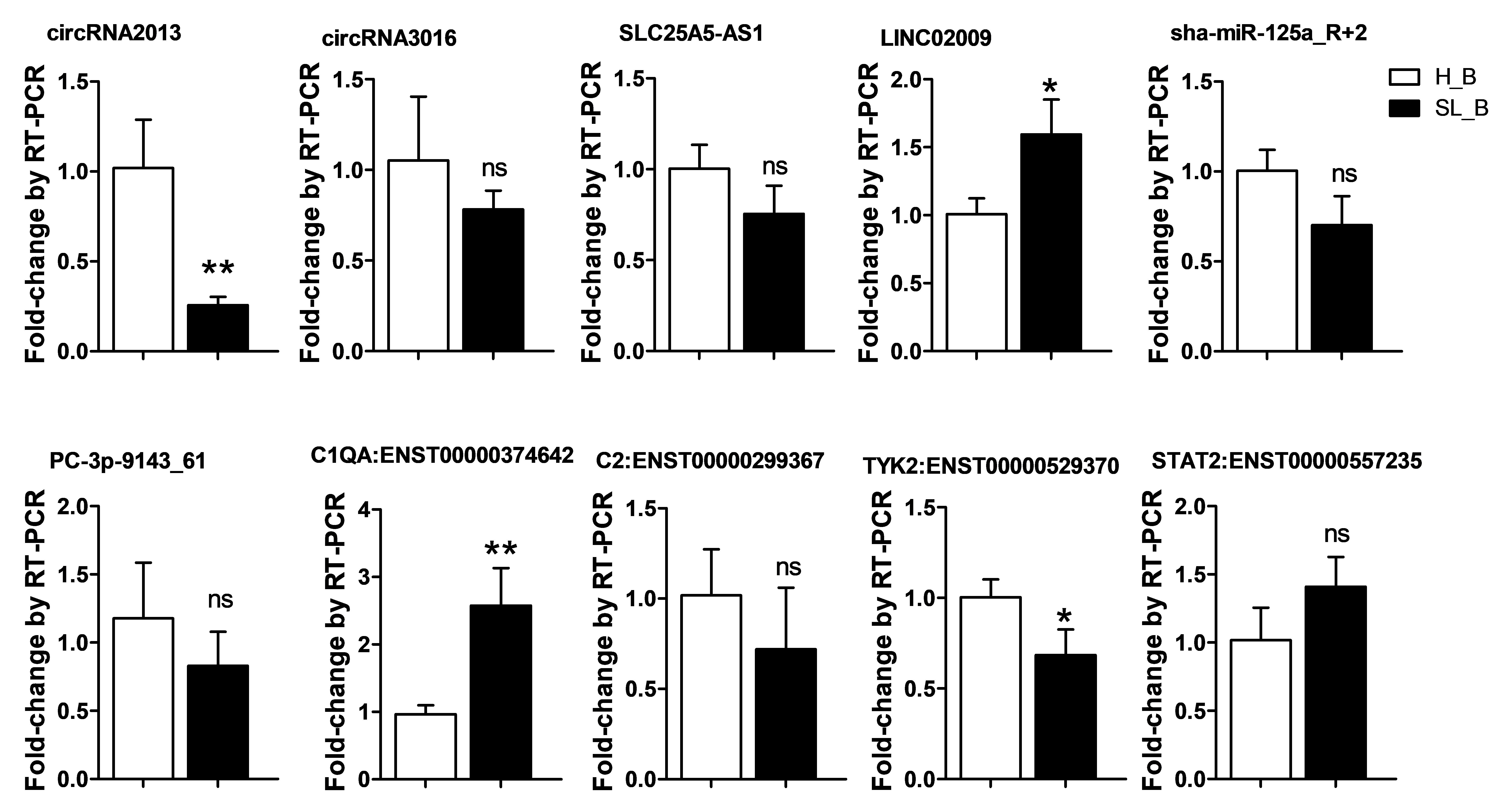

Supplement: Supplementary file 2 — Additional file 2: Figure S2. Validation of RNA sequencing data by RT-PCR. Four genes, 2 circRNAs, 2 lncRNAs and 2 miRNAs were analysed by qRT-PCR, and their relative expression levels were normalized to the housekeeping gene GAPDH or U6. Data are represented as mean ± SEM. *p < 0.05 and **p < 0 01 indicate that the differential expression of genes was significant, while ns indicates no significance (p ≥ 0.05). [file 12967_2021_3033_MOESM2_ESM.jpg]
