# Supplementary material for: Integrated analysis of competing endogenous RNA networks in peripheral blood mononuclear cells of systemic lupus erythematosus
Source: J Transl Med. 2021 Aug 21;19:362. doi: 10.1186/s12967-021-03033-8 (PMC8380341; doi:10.1186/s12967-021-03033-8)
Supplement: Supplementary file 7 — Additional file 7: Table S5. Clinical data of participants. [file 12967_2021_3033_MOESM7_ESM.docx]

**Table S5. Clinical data of participants**

| **Charateristics** | **SLE(n=40)** | **H_B(n=40)** |
| --- | --- | --- |
| Age | 35.25±8.11 | 37.80±11.10 |
| SLEDAI score, median (range) | 1(0-16) |  |
| proteinuria(g/24h) | 0.88±1.22 |  |
| ESR | 31.94±24.2 |  |
| CRP(mg/l) | 8.64±13.43 |  |
| C3(g/l) | 0.88±0.31 |  |
| C4(g/l) | 0.22±0.13 |  |
| Anti-dsDNA,n(%) | 10(50) |  |
| Anti-Sm,n(%) | 3(15) |  |
| Anti-RNP,n(%) | 7(35) |  |
| Anti-SSA/Ro, n(%) | 9(45) |  |
| Anti-SSB/La, n(%) | 2(10) |  |

SLE: systemic lupus erythematosus; H_B: healthy control; SLEDAI: SLE disease activity index; ESR: erythrocyte sedimentation rate; CRP: C reactive protein.
