# Supplementary material for: Integrated analysis of competing endogenous RNA networks in peripheral blood mononuclear cells of systemic lupus erythematosus
Source: J Transl Med. 2021 Aug 21;19:362. doi: 10.1186/s12967-021-03033-8 (PMC8380341; doi:10.1186/s12967-021-03033-8)
Supplement: Supplementary file 8 — Additional file 8: Table S6. The primers using for PCR amplification. [file 12967_2021_3033_MOESM8_ESM.docx]

**Table S6. The primers using for PCR amplification**

| NO. | Target | Sequence | TM |
| --- | --- | --- | --- |
| 1 | TYK2(NM_003331.4) | F: CAGTAGAGGAGGAGGTGAACA  R: CTGCCTTGTGAGCCTTGG | 60 |
| 2 | C1QA:ENST00000374642 | F: CAGCCTTCTCCGCCATTCG  R: CGGTTCTTCCTGGTTGGTGATG | 60 |
| 3 | C2:ENST00000299367 | F: TCTGAACCTCTACCTGCTCCT  R: GCTGAAGATCCTGTCCACCAT | 60 |
| 4 | STAT2:ENST00000557235 | F: AACCTTCAGAACCAGCAGT  R: GCCAACATAGGAGGAGAACT | 60 |
| 5 | TYK2:ENST00000529370 | F: CCTCCTTGCTTCAATCTCTT  R: ATTCATGCCATGCCAGTT | 60 |
| 6 | U6 | F: CGATACAGAGAAGATTAGCATGGC  R: AACGCTTCACGAATTTGCGT | 60 |
| 7 | sha-miR-125a_R+2 | F: TGCGTCCCTGAGACCCTAAC  RT: GTCGTATCCAGTGCAGGGTCCGAGGT ATTCGCACTGGATACGACTTTCAC | 60 |
| 8 | PC-3p-9143_61 | F: TCGGCGTAAAAACCGTGAC  RT: GTCGTATCCAGTGCAGGGTCCGAGGT ATTCGCACTGGATACGACACAGAA | 60 |
| 9 | miRNA Universal downstream primer | CAGTGCAGGGTCCGAGGTAT | 60 |
| 10 | LINC02009 | F: GCTGTTGCTCTCCGTATTGTTG  R: AGCCATCTTCCTTACACTCCAC | 60 |
| 11 | SLC25A5-AS1 | \| F: GGAGGAGGAGTCGTGGAG \| \| --- \| \| R: GGTCCAGCGTTCAAGTCC \| | 60 |
| 12 | GAPDH | F: TGCACCACCAACTGCTTAGC  R: GGCATGGACTGTGGTCATGAG | 60 |
| 13 | circRNA3016 | F: GAAGAGCCGTGAAGAACCAGATAG  R: CTCCAGACCCTTCCTCATCAGATA | 60 |
| 14 | circRNA2013 | F: GTAACTCATCCTGGCTACAT  R: GCTCTTCCAAGGGACTATT | 60 |

Note: F, forward primer; R, reverse primer; TM, annealing temperature.
