# Supplementary material for: Integrated analysis of competing endogenous RNA networks in peripheral blood mononuclear cells of systemic lupus erythematosus
Source: J Transl Med. 2021 Aug 21;19:362. doi: 10.1186/s12967-021-03033-8 (PMC8380341; doi:10.1186/s12967-021-03033-8)
Supplement: Supplementary file 10 — Additional file 10. Protocols for RNA extraction, library construction, sequencing and Bioinformatics analysis [file 12967_2021_3033_MOESM10_ESM.docx]

**Protocols for RNA extraction, library construction, sequencing and Bioinformatics analysis**

## **mRNA**

## **1.1 RNA extraction and library construction**

Total RNA was isolated and purified using TRIzol reagent (Invitrogen, Carlsbad, CA, USA) following the manufacturer's procedure. The RNA amount and purity of each sample was quantified using NanoDrop ND-1000 (NanoDrop, Wilmington, DE, USA). The RNA integrity was assessed by **Bioanalyzer 2100 (Agilent, CA, USA)** with RIN number >7.0, and confirmed by electrophoresis with denaturing agarose gel. Poly (A) RNA is purified from **2μg total RNA** using **Dynabeads Oligo (dT)25-61005 (Thermo Fisher, CA, USA)** using two rounds of purification. Then the poly(A) RNA was fragmented into small pieces using **Magnesium RNA Fragmentation Module (NEB, cat.e6150, USA)** under 94℃ 5-7min. Then the cleaved RNA fragments were reverse-transcribed to create the cDNA by **SuperScript™ II Reverse Transcriptase (Invitrogen, cat. 1896649, USA)**, which were next used to synthesise U-labeled second-stranded DNAs with E. coli DNA polymerase I (**NEB, cat.m0209, USA**), RNase H (**NEB, cat.m0297, USA**) and **dUTP Solution (Thermo Fisher, cat.R0133, USA)**. An A-base is then added to the blunt ends of each strand, preparing them for ligation to the indexed adapters. Each adapter contains a T-base overhang for ligating the adapter to the A-tailed fragmented DNA. Single- or dual-index adapters are ligated to the fragments, and size selection was performed with AMPureXP beads. After the heat-labile UDG enzyme (**NEB, cat.m0280, USA**) treatment of the U-labeled second-stranded DNAs, the ligated products are amplified with PCR by the following conditions: initial denaturation at 95℃ for 3 min; 8 cycles of denaturation at 98℃ for 15 sec, annealing at 60℃ for 15 sec, and extension at 72℃ for 30 sec; and then final extension at 72℃ for 5 min. The average insert size for the final cDNA library was 300±50 bp. At last, we performed the **2×150bp paired-end sequencing (PE150) on an illumina Novaseq™ 6000 (LC-Bio Technology CO., Ltd., Hangzhou, China)** following the vendor's recommended protocol.

## **1.2 Bioinformatics analysis of RNA-seq**

Fastp software (https://github.com/OpenGene/fastp) were used to remove the reads that contained adaptor contamination, low quality bases and undetermined bases with default parameter. Then sequence quality was also verified using fastp. We used HISAT2 (https://ccb.jhu.edu/software/hisat2) to map reads to the reference genome of *Homo sapiens* GRCh38. The mapped reads of each sample were assembled using StringTie (https://ccb.jhu.edu/software/stringtie) with default parameters. Then, all transcriptomes from all samples were merged to reconstruct a comprehensive transcriptome using gffcompare (https://github.com/gpertea/gffcompare/). After the final transcriptome was generated, StringTie and was used to estimate the expression levels of all transcripts. StringTie was used to perform expression level for mRNAs by calculating FPKM (FPKM = [total_exon_fragments / mapped_reads(millions) × exon_length(kB)]). The differentially expressed mRNAs were selected with fold change > 2 or fold change < 0.5 and with parametric F-test comparing nested linear models (p value < 0.05) by R package edgeR (https://bioconductor.org/packages/release/bioc/html/edgeR.html).

# **circRNA (rRNA removed)**

## **1.1 RNA extraction and library construction**

Total RNA was isolated and purified using TRIzol reagent (Invitrogen, Carlsbad, CA, USA) following the manufacturer's procedure. The RNA amount and purity of each sample was quantified using NanoDrop ND-1000 (NanoDrop, Wilmington, DE, USA). The RNA integrity was assessed by **Bioanalyzer 2100 (Agilent, CA, USA)** with RIN number >7.0, and confirmed by electrophoresis with denaturing agarose gel. Approximately 2μg of total RNA was used to remove ribosomal RNA according to the manuscript of the Epicentre Ribo-Zero Gold Kit (Illumina, San Diego, USA). Following purification, the ribo-minus RNA was fragmented into small pieces using **Magnesium RNA Fragmentation Module (NEB, cat.e6150, USA)** under 94℃ 5-7min. Then the cleaved RNA fragments were reverse-transcribed to create the cDNA by **SuperScript™ II Reverse Transcriptase (Invitrogen, cat. 1896649, USA)**, which were next used to synthesise U-labeled second-stranded DNAs with E. coli DNA polymerase I (**NEB, cat.m0209, USA**), RNase H (**NEB, cat.m0297, USA**) and **dUTP Solution (Thermo Fisher, cat.R0133, USA)**. An A-base is then added to the blunt ends of each strand, preparing them for ligation to the indexed adapters. Each adapter contains a T-base overhang for ligating the adapter to the A-tailed fragmented DNA. Single- or dual-index adapters are ligated to the fragments, and size selection was performed with AMPureXP beads. After the heat-labile UDG enzyme (**NEB, cat.m0280, USA**) treatment of the U-labeled second-stranded DNAs, the ligated products are amplified with PCR by the following conditions: initial denaturation at 95℃ for 3 min; 8 cycles of denaturation at 98℃ for 15 sec, annealing at 60℃ for 15 sec, and extension at 72℃ for 30 sec; and then final extension at 72℃ for 5 min. The average insert size for the final cDNA library was 300±50 bp. At last, we performed the **2×150bp paired-end sequencing (PE150) on an illumina Novaseq™ 6000 (LC-Bio Technology CO., Ltd., Hangzhou, China)** following the vendor's recommended protocol.

## **1.2 Bioinformatics analysis**

Fastp[1] was used to remove the reads that contained adaptor contamination, low quality bases and undetermined bases. Then sequence quality was also verified using fastp. We used Bowtie2[2] and Tophat2[3] to map reads to the genome of *Homo sapiens* GRCh38. Remaining reads (unmapped reads) were still mapped to genome using tophat-fusion[4]. CIRCExplorer[5-6] was used to *de novo* assemble the mapped reads to circular RNAs at first; Then, back splicing reads were identified in unmapped reads by tophat-fusion and CIRCExplorer. All samples were generated unique circular RNAs. Circular RNA expressions from different samples or groups were calculated by SRPBM = (number of back-spliced junction reads)/(number of mapped reads) × 1,000,000,000. Only the comparisons with p value<0.05 were regarded as showing differential expression by R package edgeR[7].

Reference:

1. Chen S (2018) fastp: an ultra-fast all-in-one FASTQ preprocessor. Bioinformatics

2. Langmead B, Salzberg SL (2012) Fast gapped-read alignment with Bowtie 2. Nature Methods 9: 357-359.

3. Kim D, *et al*. (2013) TopHat2: accurate alignment of transcriptomes in the presence of insertions, deletions and gene fusions. Genome Biology 14: R36.

4. Kim, D., & Salzberg, S. L. (2011). Tophat-fusion: an algorithm for discovery of novel fusion transcripts.Genome Biology , 12(8), R72

5. Zhang, X, et al. (2016). Diverse alternative back-splicing and alternative splicing landscape of circular RNAs. Genome Research, 26(9), 1277-1287.

6. Zhang, X, et al. (2014). Complementary sequence-mediated exon circularization. Cell, 159(1), 134.

7. Robinson, M. D., et al. (2010). edgeR: a bioconductor package for differential expression analysis of digital gene expression data. Bioinformatics, 26(1): 139-40.

# **lncRNA**

## **1.1 RNA extraction and library construction**

Total RNA was isolated and purified using TRIzol reagent (Invitrogen, Carlsbad, CA, USA) following the manufacturer's procedure. The RNA amount and purity of each sample was quantified using NanoDrop ND-1000 (NanoDrop, Wilmington, DE, USA). The RNA integrity was assessed by **Bioanalyzer 2100 (Agilent, CA, USA)** with RIN number >7.0, and confirmed by electrophoresis with denaturing agarose gel. Approximately 2μg of total RNA was used to remove ribosomal RNA according to the manuscript of the Epicentre Ribo-Zero Gold Kit (Illumina, San Diego, USA). Following purification, the ribo-minus RNA was fragmented into small pieces using **Magnesium RNA Fragmentation Module (NEB, cat.e6150, USA)** under 94℃ 5-7min. Then the cleaved RNA fragments were reverse-transcribed to create the cDNA by **SuperScript™ II Reverse Transcriptase (Invitrogen, cat. 1896649, USA)**, which were next used to synthesise U-labeled second-stranded DNAs with E. coli DNA polymerase I (**NEB, cat.m0209, USA**), RNase H (**NEB, cat.m0297, USA**) and **dUTP Solution (Thermo Fisher, cat.R0133, USA)**. An A-base is then added to the blunt ends of each strand, preparing them for ligation to the indexed adapters. Each adapter contains a T-base overhang for ligating the adapter to the A-tailed fragmented DNA. Single- or dual-index adapters are ligated to the fragments, and size selection was performed with AMPureXP beads. After the heat-labile UDG enzyme (**NEB, cat.m0280, USA**) treatment of the U-labeled second-stranded DNAs, the ligated products are amplified with PCR by the following conditions: initial denaturation at 95℃ for 3 min; 8 cycles of denaturation at 98℃ for 15 sec, annealing at 60℃ for 15 sec, and extension at 72℃ for 30 sec; and then final extension at 72℃ for 5 min. The average insert size for the final cDNA library was 300±50 bp. At last, we performed the **2×150bp paired-end sequencing (PE150) on an illumina Novaseq™ 6000 (LC-Bio Technology CO., Ltd., Hangzhou, China)** following the vendor's recommended protocol.

## **1.2 Bioinformatics analysis of RNA-seq**

Fastp[1] was used to remove the reads that contained adaptor contamination, low quality bases and undetermined bases. Then sequence quality was also verified using fastp. We used Bowtie2[2] and Tophat2[3] to map reads to the genome of *Homo sapiens* GRCh38. The mapped reads of each sample were assembled using StringTie [4]. Then, all transcriptome from all samples were merged to reconstruct a comprehensive transcriptome using gffcompare (https://github.com/gpertea/gffcompare/). After the ﬁnal transcriptome was generated, StringTie was used to estimate the expression levels of all transcripts. Transcripts were annotated with known mRNAs, known long non-coding RNAs (lncRNAs) and transcripts shorter than 200 nt were discarded. Then we utilized CPC [5] and CNCI [6] to predict transcripts with coding potential. All transcripts with CPC score <-1 and CNCI score <0 were removed. The remaining transcripts with class code (I, j, o, u, x) were considered as lncRNAs. StringTie was used to perform expression level for lncRNAs by calculating FPKM (FPKM = [total_exon_fragments/mapped_reads(millions) × exon_length(kB)]). The differentially expressed mRNAs and lncRNAs were selected with log2 (fold change) >1 or log2 (fold change) <-1 and with parametric F-test comparing nested linear models (p value < 0.05) by R package edgeR[7].

Reference:

1. Chen S (2018) fastp: an ultra-fast all-in-one FASTQ preprocessor. Bioinformatics

2. Langmead B, Salzberg SL (2012) Fast gapped-read alignment with Bowtie 2. Nature Methods 9: 357-359.

3. Kim D, *et al*. (2013) TopHat2: accurate alignment of transcriptomes in the presence of insertions, deletions and gene fusions. Genome Biology 14: R36.

4. Pertea M, *et al*. (2015) StringTie enables improved reconstruction of a transcriptome from RNA-seq reads. Nature Biotechnology 33: 290-295.

5. Kong L, *et al*. (2007) CPC: assess the protein-coding potential of transcripts using sequence features and support vector machine. Nucleic Acids Research 35: 345-349.

6. Sun L, *et al*. (2013) Utilizing sequence intrinsic composition to classify protein-coding and long non-coding transcripts. Nucleic Acids Research 41: e166-e166.

7. Robinson, M.D., *et al*. (2010) edgeR: a bioconductor package for differential expression analysis of digital gene expression data. Bioinformatics. 26(1), 139

# **miRNA**

## **1.1 Experimental method**

The total RNA was extracted using the Total RNA Purification Kit (LC Sciences, Houston, USA), according to the manufacturer’s protocol. The total RNA quantity and purity were analysis of Bioanalyzer 2100 and RNA 6000 Nano LabChip Kit (Agilent, CA, USA) with RIN number >7.0. Approximately 2μg of total RNA were used to prepare small RNA library according to protocol of TruSeq Small RNA Sample Prep Kits (Illumina, San Diego, USA). And then we performed the single-end sequencing (1x50bp) on an Illumina Hiseq2500 at the LC-BIO (Hangzhou, China) following the vendor’s recommended protocol.

## **1.2 Bioinformatics pipeline**

1. miRNA identification

The raw data were processed using an in-house program, ACGT101-miR (LC Sciences, Houston, Texas, USA) to remove adapter dimers, junk, low complexities, common RNA families (rRNA, tRNA, snRNA, snoRNA) (http://rfam.sanger.ac.uk/), repeats (http://www.girinst.org/repbase), and sequences <18 nt or >26 nt in length. Subsequently, the unique sequences with a length of 18~26 nt were mapped to miRNA sequences in miRBase 22.0 (http://www.mirbase.org/). Mapping was also performed on pre-miRNA against human genome GRCh38. The unique sequences that aligned to the known miRNAs sequences in miRBase 22.0 were identified as known miRNA. Secondary structure of pre-miRNAs was presented as a hairpin, including 5p- and 3p- derived miRNA. The unique sequences mapping to the other arm of the pre-miRNAs sequences, which were not annotated in the miRBase 22.0, were considered to be 5p- or 3p- derived miRNA candidates. The remaining unmapped sequences were matched to the human genomic sequences in search of candidate novel miRNAs. To identify the results of putative miRNAs in human, all the obtained miRNAs were used to predict the secondary structures using RNAfold software (http://rna.tbi.univie.ac.at/cgi-bin/RNAWebSuite/RNAfold.cgi).

1. Analysis of differentially expressed miRNAs

miRNA differential expression based on normalized deep-sequencing counts was analyzed by selectively using Fisher exact test. The significance threshold was set to be **0.05** in the test.

1. The Prediction of Target Genes to miRNAs

To predict the genes targeted by differentially expressed miRNAs, two computational target prediction algorithms (TargetScan 5.0 and miRanda 3.3a) were used to identify miRNA binding sites. Finally, the data predicted by both algorithms were combined and the overlaps were calculated. The GO terms (http://www.geneontology.org/) and KEGG Pathway (http://www.genome.jp/kegg/) of these differentially expressed miRNA targets were also annotated.
